# Neurodevelopmental outcomes and volumetric analysis of brain in preterm infants with isolated cerebellar hemorrhage

**DOI:** 10.3389/fneur.2022.1073703

**Published:** 2022-12-01

**Authors:** Seh Hyun Kim, Seung Han Shin, Hyo Ju Yang, Seul Gi Park, Soo Yeon Lim, Young Hun Choi, Ee-Kyung Kim, Han-Suk Kim

**Affiliations:** ^1^Department of Pediatrics, Seoul National University Children's Hospital, Seoul National University College of Medicine, Seoul, South Korea; ^2^Department of Radiology, Seoul National University College of Medicine, Seoul, South Korea

**Keywords:** preterm infant, cerebellar hemorrhage, brain injury, neurodevelopmental outcome, magnetic resonance imaging

## Abstract

**Background:**

Cerebellar hemorrhage (CBH) is a major form of cerebellar injury in preterm infants. We aimed to investigate the risk factors and neurodevelopmental outcomes of isolated CBH and performed volumetric analysis at term-equivalent age.

**Methods:**

This single-centered nested case-control study included 26 preterm infants with isolated CBH and 52 infants without isolated CBH and any significant supratentorial injury.

**Results:**

Isolated CBH was associated with _P_CO_2_ fluctuation within 72 h after birth (adjusted odds ratio 1.007, 95% confidence interval 1.000–1.014). The composite score in the motor domain of the Bayley Scales of Infant and Toddler Development at 24 month of corrected age was lower in the punctate isolated CBH group than that in the control group (85.3 vs. 94.5, *P* = 0.023). Preterm infants with isolated CBH had smaller cerebellum and pons at term-equivalent age compared to the control group. Isolated CBH with adverse neurodevelopment had a smaller ventral diencephalon and midbrain compared to isolated CBH without adverse neurodevelopmental outcomes.

**Conclusions:**

In preterm infants, isolated CBH with punctate lesions were associated with abnormal motor development at 24 months of corrected age. Isolated CBH accompanied by a smaller ventral diencephalon and midbrain at term equivalent had adverse neurodevelopmental outcomes.

## Introduction

Structural abnormalities of the cerebellum in premature infants can be characterized as destructive lesions with underdevelopment of the infratentorial structures. Major forms of destructive cerebellar disease include hemorrhage and infarction (1). Advances in neuroimaging techniques, such as ultrasound by mastoid fontanel view and magnetic resonance imaging (MRI) are used for diagnosing cerebellar hemorrhage (CBH), which is not an uncommon complication of preterm birth (1, 2).

There have been controversies over the neurodevelopmental outcomes of CBH based on the location and size of the lesions, such as punctate lesions in the cerebellum of preterm infants. In a retrospective study, punctate CBH was not associated with neurodevelopmental outcomes at 24 months of age (3). On the other hand, a multicenter study reported that 40% of infants with punctate CBH had abnormal developmental outcomes (4). The location of CBH is also an important determinant for neurodevelopmental outcomes as involvement of the cerebellar vermis showed global developmental delay (5, 6). As CBH often occurrs concomitant with supratentorial injury of the brain such as intraventricular hemorrhage (IVH) and periventricular leukomalacia (PVL), effect of CBH on neurodevelopmental outcomes could be biased by supratentorial lesions (7). Although there have been other studies regarding outcomes of CBH, most studies were performed with patients of CBH plus supratentorial injury (5, 8–10). Therefore, studies investigating neurodevelopmental outcomes of CBH without significant supratentorial injury are needed for better understanding of CBH in preterm infants.

Neurodevelopmental outcomes of preterm infants are not only mediated by acute destructive events of the brain but also by adverse development in regions close to and distant from the original injury (9, 11). Previous studies with volumetric analysis showed associations of reduced volume of the cerebellum and that of the total brain with poor neurological outcome at 24 months of corrected age in preterm infants (12, 13). Growth of the cerebellum is influenced by both direct damage to the cerebellum and by the growth of the contralateral cerebral hemisphere (14, 15).

In this study, we aimed to explore factors associated with isolated CBH and their neurodevelopmental outcomes and to conduct a volumetric analysis of brain structures, including the cerebellum, other infratentorial structures, and supratentorial structures of the brain, to assess the impact of isolated CBH on the volume of the cerebellum and other brain regions.

## Materials and methods

### Participants

**Figure 1:**
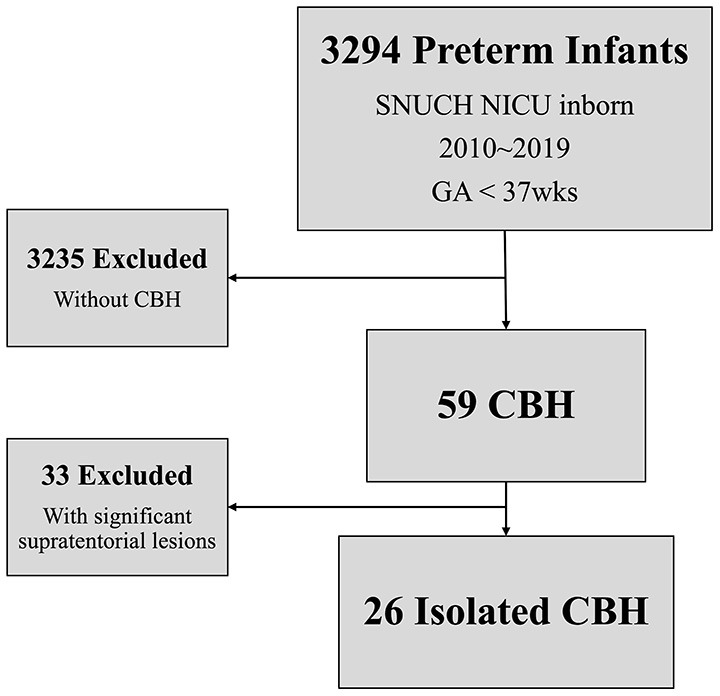
Study cohort.

This was a nested case-control study of preterm infants who were admitted to the neonatal intensive care unit (NICU) of Seoul National University Children's Hospital and ha0d brain MRI examination between January 2010 and December 2019. Infants with major congenital anomalies and significant supratentorial lesions such as severe IVH, PVL, and infarction were excluded from the study population. Isolated CBH was defined based on MRI at term equivalent age, when supratentorial lesions were not present, except for low-grade (I-II) IVH (16). Infants with isolated CBH were compared with double the number of preterm infants with gestational age (GA), birth weight, and sex-matched controls without CBH and significant supratentorial lesions to assess the risk factors and neurodevelopmental outcomes of isolated CBH. Medical records of perinatal factors, including Apgar score, preterm premature rupture of the membrane, placental abruption, histologic chorioamnionitis, chest compression in the delivery room, body temperature at admission, intubation within 3 days after birth, inotrope use within 3 days after birth, and blood gas analysis within 3 days after birth were collected. Degree of _P_CO_2_ fluctuation was calculated by subtracting the trough _P_CO_2_ level from the peak _P_CO_2_ level from results of all blood gas analysis during first 72 h life. In preterm infants, brain sonography was first conducted within 3 days after birth and subsequently performed weekly or bi-weekly according to the baby's conditions and previous sonography results during the first month of life. During the study period, there were 3,294 preterm infants admitted to Seoul National University Children's Hospital NICU between 2010 and 2019. Among them, 59 (1.8%) infants were diagnosed with CBH, with 26 (0.8%) being diagnosed with isolated CBH (Figure 1).

To evaluate the neurodevelopmental outcomes, Bayley Scales of Infant and Toddler Development 3rd Edition (BSID-III) results were gathered for each group at a corrected age (CA) of 18–24 months. On BSID-III, scores of <85 (< -1 SD) in both cognitive and language domains, or a motor score of < 85, were defined as developmental delay (17). Infants who had delay in BSID-III, hearing impairment, blindness or cerebral palsy (CP) at a CA of 18–24 months were defined as having combined neurodevelopmental impairment (NDI) (18). Hearing impairment was defined as the need for unilateral or bilateral hearing aids. Neurodevelopmental outcomes were compared between the no CBH and CBH with punctate lesion groups.

### MRI data acquisitions

The conventional neonatal brain MRI protocol includes sagittal 3d gradient echo sequence, T2- and T1-weighted axial turbo spin echo sequences, susceptibility weighted images and diffusion weighted images. T2- and T1-weighted axial turbo spin echo images and diffusion weighted images were obtained at a slice thickness of 4 mm without a gap. The sagittal 3d gradient echo sequence was obtained at a 1 mm-iso voxel and then presented as axial, coronal and sagittal reformatted images of 1 mm-thickness. Brain MRI at term-equivalent age (TEA) was routinely conducted in all preterm infants born at <29 weeks of gestation or birth weight < 1,000 g, as well as in those with parenchymal brain injury on sonography.

### MRI analysis

**Figure 2:**
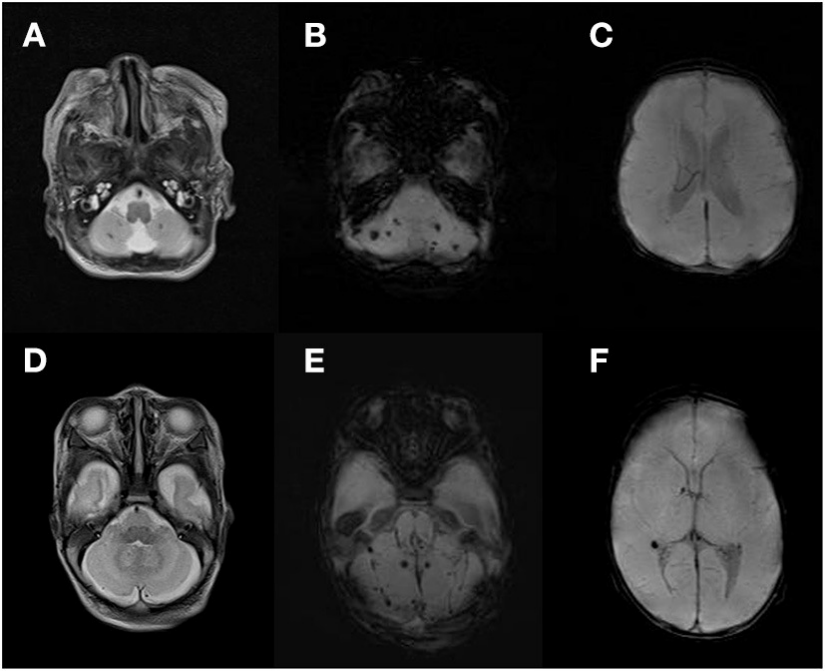
Image of isolated punctate cerebellar hemorrhage. T2 weighted image of punctate cerebellar lesion **(A,D)**. Susceptibility weighted imaging of punctate cerebellar hemorrhage **(B,E)** and supratentorial area **(C,F)**.

CBH were categorized by an experienced pediatric radiologist into four types: punctate (lesion ≤ 4 mm) CBH, limited CBH (lesion > 4 mm, involving < 1/3 of the cerebellar hemisphere), vermian hemorrhage, and massive CBH (lesion involving ≥ 1/3 of the cerebellar hemisphere), which were modified from a study by Gerda Meijler (19). Isolated CBH was defined as CBH without significant supratentorial parenchymal lesions or hydrocephalus. In present study, among 26 isolated CBH, there were 18 (69.2%) with punctate CBH (Figure 2), 7 (27.0%) with limited CBH, and 1 (3.8%) with vermian hemorrhage. No infants had massive CBH in the study population of isolated CBH.

For volumetric analysis of brain structures, the Infant Freesurfer software (https://surfer.nmr.mgh.harvard.edu/fswiki/infantFS) was used by one individual. Infant Freesurfer is a pipeline of automated segmentation and surface extraction based on Freesurfer for T1-weighted neuroimaging data for infants (20). Infant Freesurfer can calculate brain segment volumes including cerebral white matter, cerebral cortex, both cerebellar hemisphere, vermis, midbrain, pons, medulla, thalamus, hippocampus, amygdala, accumbens, and ventral diencephalon. After automatic segmentation by Infant Freesurfer, visual rechecking of each brain structure was performed. Volumetric analysis was conducted between the isolated CBH group and the control group, and then among the isolated CBH groups according to neurodevelopmental outcomes.

### Statistical analysis

Statistical analysis was executed using the R statistical software v 4.1.1. Data was expressed as number (%) or mean ± standard deviation. Wilcoxon rank-sum tests were conducted for continuous data and chi-square for categorical data. Statistical significance was set at *P* < 0.05. Univariate logistic regression analysis of GA and factors which were different between two groups was used for isolated CBH, and factors with a *P* < 0.05 in univariate analysis were inserted in the multivariate analysis.

## Results

The GA at birth (26.0 ± 2.0 vs. 26.2 ± 1.6 weeks) and birthweights (872.5 ± 587.2 vs. 867.5 ± 212.1 g) were comparable between the isolated CBH group and matched-control group (Table 1). The range of GA of both groups were 23.4–32.4 weeks. The Apgar score at 5 min was lower in the isolated CBH group than in the control group (4.5 ± 2.1 vs. 5.6 ± 2.0, *P* = 0.034). Chest compression in the delivery room, admission temperature, and intubation within 3 days after birth were comparable between the two groups. In the blood gas analysis performed within 3 days after birth, trough _P_CO_2_ levels were lower in the isolated CBH group (27.4 ± 6.5 vs. 30.8 ± 6.2 mmHg, *P* = 0.042) and _P_CO_2_ fluctuation levels (peak – trough) were higher in the isolated CBH group (37.9 ± 15.4 vs. 28.7 ± 14.7 mmHg, *P* = 0.019) (Table 2).

**Table 1:** Perinatal characteristics of study population.

	**Isolated CBH**	**Control**	***p*** **-Value**
	**(*n* = 26)**	**(*n* = 52)**	
GA (week)	26.0 ± 2.0	26.2 ± 1.6	0.683
Birthweight (gram)	872.5 ± 587.2	867.5 ± 212.1	0.966
Male	15 (57.7)	29 (55.8)	1.000
Cesarean delivery	15 (57.7)	26 (50.0)	0.689
SGA	4 (15.4)	6 (11.5)	0.905
PPROM	12 (50.0)	30 (57.7)	0.705
hCAM	10 (47.6)	32 (61.5)	0.408
Pre-eclampsia	3 (13.0)	2 (3.8)	0.332
Antenatal steroids	11 (47.8)	27 (51.9)	0.939
Multiple birth	16 (61.5)	26 (50.0)	0.47
Placental abruption	0 (0)	3 (5.8)	0.591
AS 1 min	2.5 ± 1.7	3.2 ± 2.1	0.16
AS 5 min	4.5 ± 2.1	5.6 ± 2.0	0.034
Chest compression at delivery room	6 (24)	10 (19.2)	0.855
Admission temperature (°C)	36.2 ± 1.4	36.3 ± 1.0	0.758
Intubation within 3 days	22 (91.7)	42 (80.8)	0.383
Inotrope within 3 days	6 (25.0)	10 (19.2)	0.787
IVH grade 1 or 2	12 (46.2)	22 (42.3)	0.936

CBH, cerebellar hemorrhage; GA, gestational age; SGA, small for gestational age; PPROM, preterm premature rupture of membrane; hCAM, histologic chorioamnionitis; GDM, gestational diabetes mellitus; AS, Apgar score; IVH, intraventricular hemorrhage. Values are expressed as N (%) or mean ± SD.

**Table 2:** Laboratory data of the study population.

	**Isolated CBH**	**Control**	***p*** **-Value**
	**(*n* = 26)**	**(*n* = 52)**	
**pH**			
Trough within 3 days	7.1 ± 0.1	7.2 ± 0.1	0.088
Peak within 3 days	7.4 ± 0.1	7.4 ± 0.1	0.3
_**P**_**CO**_**2**_ **(mmHg)**			
Trough within 3 days	27.4 ± 6.5	30.8 ± 6.2	0.042
Peak within 3 days	65.3 ± 13.7	59.5 ± 14.9	0.122
Fluctuation (peak-trough)	37.9 ± 15.4	28.7 ± 14.7	0.019
**Base excess**			
Trough within 3 days	−11.6 ± 5.3	−9.7 ± 3.0	0.14
Cord Magnesium (mg/dl)	2.8 ± 1.0	3.0 ± 1.1	0.508

CBH, cerebellar hemorrhage; _P_CO_2_, partial pressure of carbon dioxide. Values are expressed as N (%) or mean ± SD.

Univariate regression analysis showed that 5 min Apgar score and _P_CO_2_ fluctuation level within 3 days after birth were associated with isolated CBH (Table 3). Multivariate logistic regression showed that the _P_CO_2_ fluctuation level was significantly associated with isolated CBH [adjusted odds ratio (OR) 1.007, 95% confidence interval (CI) 1.000–1.014, *P* = 0.044), while the association with 5 min Apgar score became insignificant (adjusted OR 0.961, 95% CI 0.914–1.011, *P* = 0.126).

**Table 3:** Univariate and multivariate logistic regression analyses for factors related to isolated CBH.

	**OR**	**95% CI**	***p*** **-Value**	adjOR^*^	**95% CI**	***p*** **-Value**
GA	0.988	[0.928, 1.051]	0.683	0.994	[0.937, 1.054]	0.570
AS 5 min	0.947	[0.901, 0.996]	0.034	0.961	[0.914, 1.011]	0.126
_P_CO_2_ Fluctuation within 3 days	1.008	[1.002, 1.015]	0.019	1.007	[1.000, 1.014]	0.044

* Adjusted for GA, AS 5 min, and delta _P_CO_2_ within 3 days. OR, odds ratio; CI, confidence interval; GA, gestational age; AS, Apgar score; CBH, cerebellar hemorrhage; _P_CO_2_, partial pressure of carbon dioxide.

In terms of neurodevelopmental outcome, there were no cases of CP, hearing loss and blindness in the study population and overall NDI was comparable between the isolated punctate CBH and matched-control groups. The motor domain in the BSID showed lower scores in the isolated punctate CBH group (85.3 ± 10.3 vs. 94.5 ± 11.5, *P* = 0.023) (Table 4).

**Table 4:** Neurodevelopmental outcomes at 18–24 months corrected age.

	**Punctate isolated CBH**	**Control**	***p-*** **Value**
	**(*n* = 18)**	**(*n* = 52)**	
Neurodevelopmental impairment	6 (33.3)	7 (13.5)	0.129
Cerebral palsy	0 (0)	0 (0)	1.000
Hearing loss	0 (0)	0 (0)	1.000
Blindness	0 (0)	0 (0)	1.000
BSID-III	*n* = 11	*n* = 32	
Cognitive domain	88.6 ± 11.9	94.4 ± 15.0	0.258
Language domain	85.6 ± 13.2	91.5 ± 17.0	0.303
Motor domain	85.3 ± 10.3	94.5 ± 11.5	0.023
Cognitive and language < 85	2 (18.2)	5 (15.6)	1.000
Motor < 85	6 (54.5)	6 (18.8)	0.058

CBH, cerebellar hemorrhage; BSID-III, Bayley Scales of Infant and Toddler Development 3rd edition. Values are expressed as N (%) or mean ± SD.

In the volumetric analysis of the brain structures, the isolated CBH group showed smaller cerebellum (11.7 ± 3.0 vs. 13.5 ± 3.0 ml, *P* = 0.007) and pons (1.5 ± 0.4 vs. 1.8 ± 0.3 ml, *P* = 0.001) volume compared to the control group. The estimated total intracranial and vermian volumes were comparable between the two groups (Table 5). In the subgroup analysis of isolated CBH, those with NDI had smaller ventral diencephalon (1.5 ± 0.2 vs. 1.7 ± 0.2 ml, *P* = 0.028) and midbrain (1.3 ± 0.2 vs. 1.4 ± 0.1 ml, *P* = 0.032) than those without NDI at CA 18–24 months (Table 6).

**Table 5:** Volumetric MRI analysis of brain structures of the study population.

	**Isolated CBH**	**Control**	***p*** **-Value**
	**(*n* = 26)**	**(*n* = 52)**	
GA (week)	26.0 ± 2.0	26.2 ± 1.6	0.683
Birthweight (gram)	872.5 ± 587.2	867.5 ± 212.1	0.966
PMA at MRI (week)	37.6 ± 2.2	36.8 ± 1.4	0.107
Body weight at MRI (g)	2,192.7 ± 365.4	2,319.4 ± 383.2	0.166
Head circumference at MRI (cm)	30.9 ± 2.1	30.9 ± 1.9	0.895
Left cerebellar hemisphere (ml)	5.1 ± 1.4	6.1 ± 1.3	0.003
Right cerebellar hemisphere (ml)	5.3 ± 1.3	6.0 ± 1.4	0.062
Vermis (ml)	1.2 ± 0.4	1.4 ± 0.5	0.065
Cerebellum (ml)	11.7 ± 3.0	13.5 ± 3.0	0.007
Midbrain (ml)	1.4 ± 0.2	1.4 ± 0.2	0.059
Pons (ml)	1.5 ± 0.4	1.8 ± 0.3	0.001
Medulla (ml)	0.9 ± 0.3	0.9 ± 0.2	0.367
Ventral diencephalon (ml)	1.6 ± 0.2	1.7 ± 0.2	0.111
Accumbens area (ml)	0.5 ± 0.1	0.5 ± 0.1	0.927
Amygdala (ml)	0.9 ± 0.2	0.9 ± 0.2	0.991
Pallidum (ml)	1.6 ± 0.3	1.7 ± 0.4	0.376
Putamen (ml)	3.5 ± 0.8	3.6 ± 0.7	0.637
Caudate (ml)	2.4 ± 0.5	2.6 ± 0.5	0.116
Hippocampus (ml)	1.6 ± 0.2	1.7 ± 0.2	0.113
Thalamus (ml)	8.4 ± 1.4	8.7 ± 1.1	0.297
Cerebral white matter (ml)	89.9 ± 14.4	96.3 ± 14.4	0.07
Total intracranium (ml)	256.5 ± 50.5	266.2 ± 42.4	0.372

CBH, cerebellar hemorrhage; GA, gestational age; PMA, postmenstrual age. Values are expressed as N (%) or mean ± SD.

**Table 6:** Volumetric MRI analysis of isolated CBH with and without NDI.

	**Isolated CBH with NDI**	**Isolated CBH without NDI**	***p*** **-Value**
	**(*N* = 9)**	**(*N* = 17)**	
GA (week)	24.9 ± 1.4	26.6 ± 2.0	0.037
Birthweight (gram)	706.7 ± 174.7	960.4 ± 707.0	0.177
Left cerebellar hemisphere (ml)	4.7 ± 1.2	5.3 ± 1.4	0.279
Right cerebellar hemisphere (ml)	5.0 ± 1.1	5.5 ± 1.5	0.368
Vermis (ml)	1.0 ± 0.2	1.3 ± 0.5	0.079
Cerebellum (ml)	10.8 ± 2.4	12.2 ± 3.2	0.258
Midbrain (ml)	1.3 ± 0.2	1.4 ± 0.1	0.032
Pons (ml)	1.3 ± 0.4	1.6 ± 0.4	0.072
Medulla (ml)	0.9 ± 0.2	0.9 ± 0.3	0.991
Ventral diencephalon (ml)	1.5 ± 0.2	1.7 ± 0.2	0.028
Accumbens area (ml)	0.5 ± 0.1	0.4 ± 0.1	0.557
Amygdala (ml)	0.8 ± 0.1	0.9 ± 0.2	0.291
Pallidum (ml)	1.6 ± 0.2	1.7 ± 0.3	0.325
Putamen (ml)	3.4 ± 0.6	3.6 ± 0.8	0.463
Caudate (ml)	2.3 ± 0.5	2.5 ± 0.5	0.438
Hippocampus (ml)	1.5 ± 0.3	1.7 ± 0.2	0.148
Thalamus (ml)	7.9 ± 1.4	8.6 ± 1.4	0.252
Cerebral white matter (ml)	85.4 ± 12.8	92.3 ± 15.0	0.259
Total intracranium (ml)	256.3 ± 58.6	256.7 ± 47.6	0.984

CBH, cerebellar hemorrhage; NDI, neurodevelopmental impairment; GA, gestational age. Values are expressed as N (%) or mean ± SD.

## Discussion

The current study showed that preterm infants with isolated CBH had more fluctuations in _P_CO_2_ levels within 72 h after birth than those without CBH. Isolated CBH with punctate hemorrhage is associated with abnormal motor development in preterm infants. Among preterm infants with isolated CBH, the ventral diencephalon and midbrain were smaller in those with NDI than in those without NDI. The incidence of CBH and isolated CBH in the present study was 1.8 and 0.8%, respectively, which were consistent with previous studies that reported incidence ranges of CBH in preterm infants from 1.5 to 19%, and the incidence of isolated CBH from 0.9 to 5.2% depending on the imaging modality (2, 10, 21).

CBH is known to be more prevalent in smaller and more immature preterm infants, and several factors have been reported as risk factors such as preeclampsia, sepsis, prolonged mechanical ventilation, patent ductus arteriosus, lower Apgar scores, acidosis, and hypotension (8, 22–25). In the present study, we investigated the risk of isolated CBH, wherein fluctuation in _P_CO_2_ within 72 h after birth was the only factor associated with isolated CBH. Impaired autoregulation of the cerebral circulation in preterm infants is associated with CBH (4, 26). As hypercapnia alters cerebral hemodynamics by increasing blood flow and on the contrary, low _P_CO_2_ has vasoconstrictive effects (27, 28). These effects of _P_CO_2_ that alter cerebral hemodynamics might explain the association of isolated CBH with higher _P_CO_2_ fluctuation in the present study. Thus, careful ventilator strategy during the first 3 days after birth to avoid over- or under-ventilation might be helpful to avoid CBH.

The cerebellum undergoes exponential growth such that the volume expands 5-fold from 24 weeks to 40 weeks of gestation, and this growth rate is faster than any other structures of the human brain (1). Due to this exponential growth, the cerebellum of a premature infant is vulnerable to multiple insults, and large CBH are associated with poorer neurodevelopmental outcomes and cerebellar injury is associated with various degree of motor delay (1, 25). However, there is a limited understanding of the effect of CBH on neurodevelopment in preterm infants because CBH is often accompanied by supratentorial injuries of brain in this population. A systematic review by Hortensius et al. contributed to expanding the understanding of this problem to some extent and reported that isolated CBH in preterm infants was associated with neurodevelopmental delay in the cognitive, language, behavioral, and motor domains, with the highest incidence of vermian involvement and CBH with large hemorrhages (10).

Punctate hemorrhage accounts for 39–67% of isolated CBH in preterm infants (4, 29). Size of hemorrhage in CBH is one of the important determents for prognosis, as large CBH had higher incidence of motor impairment (10). These small punctate CBH are usually not found on head ultrasounds but are often diagnosed on TEA brain MRI (4). And punctate CBH are known to have more favorable neurodevelopmental outcomes (3, 4). In a retrospective study, CBH with punctate lesions was not associated with adverse neurodevelopment (3). However, the study included preterm infants with supratentorial lesions, which might mask the effect of isolated CBH. On the contrary, in a systematic review of isolated CBH, 13% of the patients with punctate CBH had significant neurodevelopmental impairment (10). The present study showed that punctate isolated CBH was associated with poorer motor function, showing NDI in 33% and BSID-III motor scores below 85 in 55% among isolated CBH with punctate lesion. However, there was no difference in the incidence of CP between the isolated CBH group and the control group. Distinguishing punctate cerebellar hemorrhage from the simple deposition of circulating blood in the cerebrospinal fluid is not simple. Disproportional deposition of hemosiderin in the posterior fossa, accompanied parenchymal hemosiderin deposits in the susceptibility weighted imaging, and parenchymal change such as atrophy might be helpful to distinguish the condition.

Decreased cerebellar volume is associated with poor neurodevelopmental outcomes, which could be mediated by effects on adverse development in regions close to and remote from the original injury (9, 11). The present study demonstrated that the isolated CBH group had smaller cerebellar and pons volumes than the control group. In a previous study, cerebellar atrophy developed after hemorrhages or infarcts with a prevalence of 20–37% in infants with CBH (2, 8). In addition, smaller pons was associated with CBH in premature infants in a case series by Parodi et al., which showed that unilateral CBH and crossed pontine hemiatrophy were related (30). In a MRI study that compared volumetric analysis of *in-utero* and *ex-utero* brain of term and preterm infants, volume of cerebellar hemisphere and pons at TEA were smaller in preterm infant than term infants (31).

Among preterm infants with isolated CBH, the ventral diencephalon and midbrain were smaller in the isolated CBH with NDI group than in the isolated CBH without NDI group. The ventral diencephalon is a combination of several structures, including the basal forebrain, ventral tegmentum, and hypothalamus. The midbrain connects the cerebellum, pons with the forebrain and the ventral midbrain, which contains the pyramidal and corticopontine tracts, which are related to motor functions (32). Also, the ventral tegmentum is known to play an important role in dopamine-related functions, including motor functioning (33). As shown in the present study, if isolated CBH is accompanied by a reduced volume of specific brain structures, including the ventral diencephalon and midbrain, the probability of developmental impairment might increase. This was also well-demonstrated in the study by Limperopoulos et al., which reported that neurodevelopmental impairment in preterm infants with cerebellar injury was accompanied by remoted growth restriction of cerebral cortical growth (34).

The current study had several limitations. This was a retrospective study, and brain MRI was performed in the selected high-risk population of preterm infants. In addition, only two-thirds of the infants were evaluated by Bayley-III at 18–24 months of CA, although data regarding CP, hearing loss and blindness were well-collected. Those who was not tested by Bayley-III among the study population were those whose parents thought that their child had normal development during the follow-up period. In addition, there is a possibility that some punctate CBH lesions may not be visible on TEA brain MRI in the study population and this may influence the results. However, the risk factors, neurodevelopmental outcomes, and brain structure volumetric analysis of isolated CBH were thoroughly analyzed. Volumetric MRI analysis of isolated CBH with or without NDI was also performed. This retrospective case-control study suggests that isolated CBH is associated with higher fluctuations in _P_CO_2_ levels within 72 h after birth. Additionally, when isolated CBH was combined with a reduced volume of the ventral diencephalon and midbrain, poorer neurodevelopmental outcomes were observed. Further studies with larger populations are required to clarify the neurodevelopmental outcomes of isolated CBH.

## Data availability statement

The original contributions presented in the study are included in the article/supplementary material, further inquiries can be directed to the corresponding author.

## Ethics statement

The studies involving human participants were reviewed and approved by Seoul National University Hospital Institutional Review Board. Written informed consent from the participants' legal guardian/next of kin was not required to participate in this study in accordance with the national legislation and the institutional requirements.

## Author contributions

SK interpreted the collected MRI data, performed statistical analysis, and wrote the initial draft of the manuscript. HY, SP, and SL interpreted and collected clinical data. SS and YC developed the study idea, performed statistical analysis, and critically reviewed the manuscript. E-KK and H-SK critically reviewed the manuscript. All authors gave substantial contributions to the work, drafted, revised, reviewed, approved of the final manuscript, and agreed to be accountable for all aspects of the work.

## Funding

This research was supported and funded by Seoul National University Hospital Kun-hee Lee Child Cancer and Rare Disease Project, Republic of Korea (Grant Number: 22C-014-0100).

## Conflict of interest

The authors declare that the research was conducted in the absence of any commercial or financial relationships that could be construed as a potential conflict of interest.

## Publisher's note

All claims expressed in this article are solely those of the authors and do not necessarily represent those of their affiliated organizations, or those of the publisher, the editors and the reviewers. Any product that may be evaluated in this article, or claim that may be made by its manufacturer, is not guaranteed or endorsed by the publisher.
